# Non-Suicidal Self-Injury (NSSI) Patterns in Adolescents from a Romanian Child Psychiatry Inpatient Clinic

**DOI:** 10.3390/children11030297

**Published:** 2024-03-01

**Authors:** Lucia Emanuela Andrei, Magdalena Efrim-Budisteanu, Ilinca Mihailescu, Alexandra Mariana Buică, Mihaela Moise, Florina Rad

**Affiliations:** 1 Child and Adolescent Psychiatry Department, Carol Davila University of Medicine and Pharmacy, 020021 Bucharest, Romania; lucia.andrei@drd.umfcd.ro (L.E.A.); ilinca.mihailescu@umfcd.ro (I.M.); alexandra.buica@umfcd.ro (A.M.B.); mihaela.stancu@drd.umfcd.ro (M.M.); florina.rad@umfcd.ro (F.R.); 2 Child and Adolescent Psychiatry Department, “Prof. Dr. Al. Obregia” Clinical Hospital of Psychiatry, 041914 Bucharest, Romania; 3 Faculty of Medicine, “Titu Maiorescu” University, 040441 Bucharest, Romania

**Keywords:** Non-Suicidal Self-Injury, self-harm, adolescent, depression, NSSI-AT, suicide, personality

## Abstract

Non-Suicidal Self-Injury (NSSI) involves deliberately causing harm to one’s body without the intention of suicide. As the numbers of adolescents presenting NSSI have been steadily increasing during the last years, we intended to investigate adolescent patients exhibiting NSSI, admitted to our clinic—a Romanian child psychiatry inpatient clinic, over the course of five years. A total of 100 adolescents (80 females, 20 males, mean age: 14.9 years) hospitalized for various neuropsychiatric disorders and engaging in self-harm were studied. The self-harm methods most frequently used in our sample were, for the female group: cutting (all), skin tearing (76%), scratching/pinching (72%), and for males: cutting (all), wound-healing hindrance (85%), striking objects (80%). The initial motivations for NSSI were represented by distress (females 89%, males 90%) and seeking pleasure (females 84%). In terms of the roles of NSSI, it was primarily used for emotional regulation (females 89%) and anger management (males 90%). This study highlights the prevalence of self-harm in hospitalized adolescents, differences in methods and motivations between genders, and the need for more targeted therapy interventions. By documenting trends, investigating underlying motivations and functions, and proposing hypotheses for further research, our findings offer valuable insights on adolescent NSSI and have the potential to increase awareness among various clinicians and specialists who interact with adolescents, thus addressing the escalating prevalence of self-harm behaviours among teenagers.

## 1. Introduction

Non-Suicidal Self-Injury (NSSI) refers to deliberate self-injurious behaviour to one’s body, with the assumption that the behaviour will not lead to major physical harm and with no suicidal intent [1,2,3]. The introduction of NSSI in the DSM-5 (5th version of the Statistical and Diagnostic Manual of Mental Disorders) for the first time as a “research diagnosis requiring further study” under section III reflects the rising interest in the phenomenon, both from a clinical, as well as a research perspective [1,4]. The prevalence of these behaviours has been studied in both clinical and non-clinical populations. Current data indicate that throughout their lives, an estimated 13% to 17% of young people experience self-harm [5], while other studies found that almost 50% of adolescents from in-patient samples engage in NSSI [6,7]. Some studies analysing the age of onset of this phenomenon report that NSSI starts between the ages of 11 and 15 [8], while other studies found that NSSI has a peak in adolescence between 15–17 years old, with progressive remission during early adulthood [9]. Data from meta-analyses show that across development, among younger adolescents, the frequency of NSSI is on the rise. This frequency stabilizes in middle adolescence, before declining in older adolescents [10]. NSSI methods vary considerably from one teenager to another, the most frequent reported practices represented being cutting, hitting, scratching and burning oneself [11].

Emotional regulation, self-punishment, and distress communication are described as the most common reasons that lay behind these behaviours by several studies using patients’ self-reports [12,13]. The underlying causes of self-harm have been a matter of high interest in several reviews on the empirical research about this phenomenon, as the authors tried to identify the effects of this behaviour on affective arousal. As per the information we have so far on the matter, it is considered that those who partake in these behaviours manifest deficits in emotional regulation. Thus, emotional regulation has been defined as a major function of NSSI [7,8,9,11,12,13]. Emotional regulation is defined as a complex construct that includes emotional awareness, understanding and acceptance, impulsive behaviour inhibition abilities in relation to emotional distress, as well as the willingness to avoid activities that may trigger negative emotions [10]. As NSSI is frequently done so that the perception of negative affect is decreased, studies suggest it is a common strategy for regulating emotions [14].

As the number of patients who resort to NSSI has been increasing in the last years, there has been a constant need to develop adequate assessment instruments for these behaviours. Nevertheless, the current available research tools for assessing NSSI are still scarce or not taking into account all relevant aspects of NSSI when evaluating suicide risk. The NSSI-AT (NSSI-Assessment Tool) has demonstrated effectiveness in uncovering the biological, psychological, and social factors underlying NSSI. Since diagnostic measures for NSSI are limited, exploring its broader psychosocial dimensions can aid understanding and intervention [2,15].

Adolescence is a period of significant physical, emotional, and cognitive development. Adolescents may engage in self-harm as a way to cope with the challenges and stressors associated with this developmental stage, such as identity formation, peer relationships, academic pressure, and family conflicts. Studying self-harm in adolescents provides insights into how these developmental factors intersect with self-harming behaviours. Self-harming behaviours during adolescence can have significant long-term consequences for mental health, well-being, and functioning. Adolescents who engage in self-harm are at increased risk of developing mental health disorders, substance abuse problems, and engaging in suicidal behaviours later in life. Understanding the factors contributing to self-harm in adolescence can inform early intervention efforts aimed at preventing these negative outcomes [16].

In this study, we aimed to characterize patients admitted to a Romanian child and adolescent psychiatry clinic over a period of 5 years who presented with NSSI, focusing on NSSI aspect, severity, motivation, together with practiced patterns of self-harm and impact on personal, school and social functioning, using the NSSI-AT. Our intention is to provide valuable insights into the prevalence rates of self-harming behaviours among a specific population, namely psychiatric adolescent patients aged 13–17 years. Additionally, shedding light on NSSI would offer insights that can inform prevention and intervention strategies.

Through the analysis of demographic and environmental data from our patients, our study aims to highlight some risk factors that heighten susceptibility to self-harm. This understanding can guide the development of targeted support strategies for individuals vulnerable to self-harm.

Furthermore, our study, considering the evaluation instrument and methods utilized, has the potential to enhance the understanding of self-harming behaviours among clinicians from various medical specialties focusing on the health of children and adolescents. This improved understanding facilitates more accurate assessment and timely diagnosis of such conditions, leading to the development of tailored treatment plans and the provision of appropriate support and care for individuals experiencing self-harm. Such interventions could encompass psychoeducation programs, skill-building workshops, cognitive-behavioural therapies, and improved availability of mental health services. These efforts are directed towards minimizing self-harm and fostering the adoption of healthier coping strategies.

## 2. Materials and Methods

### 2.1. Participants

We conducted an analytical, non-experimental, descriptive study on 100 adolescents (age range 13–17) admitted in the Child and Adolescent Psychiatry Department at Prof. Dr. Al. Obregia Clinical Psychiatry Hospital in Bucharest, between January 2019–November 2023 who presented with NSSI.

The age range was selected for two reasons: firstly, due to the fact that the clinic in which the research was conducted is dedicated to children and adolescents under 18 years of age and, secondly, as the majority of those who struggle with these behaviours and are referred to the aforementioned clinic are adolescents aged 13–17 years. They were also generally more inclined to cooperate and take part in the study. Moreover, current research indicates that rates of self-harm tend to peak during adolescence, making this population a critical group to study in order to understand the scope and nature of the phenomenon.

The patients were approached regarding inclusion in this study by one of the physicians involved in the research, during the course of their hospitalization in the clinic and were informed regarding the evaluation process and methods. They were included in the study after their agreement to participate, as well as the agreement of their legal guardian—expressed by signing the informed consent. In the period of time during which this research was conducted, a total of 911 adolescent patients presenting with NSSI were identified. To have a confidence level of 95%, a sample size of 271 was needed. However, the final group consisted of 100 subjects—thus, with a margin of error of 9.06%, as 100 represents the total number of patients that agreed to take part in the research.

Through the non-experimental design of this study, the subjects’ participation involved no risk to their physical or mental health. All the collected patient data were registered under individualized patient codes, in order to maintain participant confidentiality. The selection criteria for participants and our research methodology were implemented with the approval of the Ethics Committee at the Prof. Dr. Alexandru Obregia Clinical Psychiatry Hospital.

Each participant underwent an extensive psychiatric evaluation conducted by a psychiatrist, which included gathering medical and developmental histories, demographical data, educational backgrounds, and conducting a mental status examination.

In the studied group, the main psychiatric diagnosis was represented by a Depressive Disorder (DD). The DDs and other comorbid psychiatric diagnoses were established by a child and adolescent psychiatrist based on ICD-10 (International Classification of Diseases, 10th Revision) criteria and confirmed by psychiatric structured interviews. Socio-demographic and clinical data were recorded in a database [17].

This study excluded patients with a current diagnosis of severe depressive episode with psychotic symptoms or an intellectual disability (IQ < 70). We excluded patients with psychotic disorders and/or intellectual disability due to these diagnoses’ potential to significantly impact cognitive functioning and thought processes. Including individuals with these conditions could have introduced confounding variables that might have obscured the study’s results or made it challenging to interpret them accurately. Moreover, we considered it ethically appropriate to avoid subjecting such vulnerable patients to potential distress.

### 2.2. Instruments

The NSSI-AT (Non-Suicidal Self-Injury Assessment Test) designed by J. Whitlock and A. Purington at Cornell University [2,15] was used in order to assess the non-suicidal self-harm behaviours. The NSSI-AT is a tool created to evaluate NSSI, aiming to examine both primary aspects (such as form, frequency, and function) and secondary NSSI features (which encompass but are not limited to NSSI habituation, contexts of NSSI occurrence, and perceived life interference, treatment, and impacts related to NSSI). The test does not provide a total score or a cut-off. The findings presented by the assessment tool authors endorse the reliability of NSSI-AT test scores, assessed through test-retest analysis, as well as the validity of NSSI-AT test score interpretations for the behaviour and frequency modules. This validity was assessed through concurrent, convergent, and discriminant evidence in the population under scrutiny and is suitable for use with clinical samples of adolescents.

For our clinical research, an adapted NSSI-AT Romanian version from Whitlock and A. Purington was used, after the approval of Dr. J. Whitlock. In the translation process, great efforts were made to maintain the original meaning of each item of the tool; however, we argue there could be some potential limitations due to cultural biases and language differences.

All participants underwent the administration of the NSSI-AT, administered as a paper-and-pencil assessment, by a physician. The questionnaire consists of 39 items, organized into 12 modules developed to assess several NSSI characteristics. As per the structure of the questionnaire (presence of items that include both quantitative and qualitative responses) and in accordance with the aims of our study, we considered only the following NSSI-AT items: Primary and secondary NSSI characteristics; Functions; Recency and Frequency; Wound Locations; Initial Motivations; Severity; Practice Patterns; Perceived Life Interference; NSSI Treatment Experiences [15]. However, we acknowledge that our option to focus specifically on these particular NSSI-AT items could influence the study’s findings.

In this descriptive study, we analysed patterns of behaviour in teenagers who engage in NSSI, by using the NSSI-AT instrument on a clinical sample. The used method could have several implications on the validity of the results, that reside in the subjective nature of the participants’ answers—which could be influenced by factors such as their psychiatric comorbidities or their potential tendencies to either exacerbate or diminish the severity or other characteristics of the self-harming habits, as well as the fact that in a clinical (hospitalized) population sample, various aspects of these behaviours could be significantly different in comparison to the general population.

### 2.3. Statistical Analysis

A retrospective analysis of all subjects’ records was conducted using the clinic’s database. This analysis investigated the characteristics, severity, and dynamic aspects of their diagnoses, as well as the number and consistency of any comorbid psychiatric conditions. All variables were subjected to descriptive analysis for the socio-demographic and clinical data; mean and standard deviation (SD) was calculated for the variables: patients’ age and age of onset of self-harming behaviours.

Compilation of the variables database was carried out with Microsoft Office Excel 2007. Descriptive analysis of the qualitative and quantitative variables was performed.

## 3. Results

### 3.1. Socio-Demographic Characteristics

Table 1 presents the sample’s sociodemographic characteristics. In our sample of 100 adolescent patients hospitalized for various acute and chronic neuropsychiatric disorders and selected for engaging in self-harming behaviours, 80 were females and 20 males, with a mean age of 14.9 years (SD ± 1.51). The mean age at NSSI onset, determined from NSSI-AT, was 14.72 years (SD ± 1.87) for females and, 15.2 years (SD ±1.72) for male subjects, female patients reporting an earlier onset of self-harming behaviours. Eighty-five of our patients (63 female, 12 male) declared living in an urban area, while twenty-five of them (17 female, 8 male) came from a rural setting.

### 3.2. Psychiatric Comorbidities

The Clinical diagnoses of the study group are shown in Table 2. In the selected patient sample, a diagnosis of a DD (Depressive Disorder) was identified in the whole group, while the second most frequent psychiatric comorbidity was one from the anxiety disorders range which included obsessive-compulsive disorders. These diagnoses have been identified predominantly in the female group, with 59% of girls being diagnosed with and anxiety disorder, in comparison to 35% of boys having such difficulties. A diagnosis of conduct disorder has been identified in 16% of our female respondents and 20% of our male subjects. None of the male participants were diagnosed with an eating disorder, while 14% of girls had this diagnosis. An important aspect when it comes to the psychiatric comorbidities of our sample (adolescents aged 13–17 years old) is the disharmonious personality traits that were identified in the majority of our respondents (85% of girls, and 80% of boys). We used the term “disharmonious personality traits” referring to traits or characteristics that are not in alignment or congruent with one another, causing internal conflict or inconsistency within an individual’s personality. These traits may manifest as conflicting patterns of behaviour, emotions, or thoughts that create tension or discord within the individual’s psyche. Disharmonious personality traits (such as ambivalence, impulsivity, perfectionism, passive-aggressiveness, rigidity, grandiosity, insecurity and dependency) are often viewed as maladaptive or problematic because they can hinder personal development, impair social functioning, and contribute to psychological distress. This term was used considering that an individual’s personality is still developing through adolescence and thus, a diagnosis of a personality disorder is not clearly defined during this period.

### 3.3. Characteristics of NSSI

As it is illustrated in Table 3, in terms of self-injury forms, the methods that were mostly used by our female patients were cutting (of wrists, arms, legs, torso or other areas of the body)—all of the female patients declaring that at some point they used cutting as a self-harming method, followed by skin tearing (76%) and severe “scratching or pinching, resulting in bleeding or lasting marks on the skin” (72%) [15]. In the male group, the cutting method was used by 100% of them as well, while deliberately hindering the healing process of wounds was the second most used self-injury method (85% of boys), followed by striking or impacting objects until bruising or bleeding occurred, as 80% male respondents engaged in such behaviours.

Table 4 presents Self-injury functions in our group. The function of the self-harming behaviours has been the focus of many studies done on NSSI. According to the NSSI research, the functions of these behaviours are divided into 5 categories: affective imbalance, low pressure, social communication and expression, self-retribution and deterrence, sensation seeking, and last but not least—affective imbalance, high pressure. Of these categories, the majority of the female respondents (89%) declared using self-harm as a means of regulating affective imbalance, in order to “to feel something”. In the boys’ group, the main purpose of self-injurious behaviours was “to deal with anger”, categorized as an affective imbalance, high pressure situation.

Recency and frequency of NSSI are shown in Table 5.

“Recency” was used to evaluate the most recent occasion the patients intentionally harmed themselves. The majority of patients in our group reported that they engaged in self-injurious actions less than a month prior to the moment of the investigation, with 89% of female and 85% of male respondents affirming this. In terms of behaviour frequency, 64% girls reported engaging in self-harm 2–10 times at the time of the study, while 45% declared engaging in self-harm between 11–50 times.

Table 6 illustrates Wound locations found in our group. In terms of wound locations, all of our patients, both male and female, reported the wrists as main location for performing self-harm on, followed by their arms (91% of girls and 75% of boys), and calves or ankles (65% of boys and 59% of girls).

The initial motivation expressed by the study participants is shown in Table 7. The motivation behind the initiation of such behaviours was a multimoded concept for our patients, as they all declared multiple reasons that were behind the starting of the NSSI. Thus, in the case of girls, the main motivation to self-harm was represented by their deciding to try it while they were feeling distressed (89%), closely followed by seeking a pleasurable sensation (84%). Similarly, in the male group, deciding to try it while they were feeling distressed (90%) was the main motivation to engage in self-harming actions.

Table 8 shows the NSSI severity. In terms of severity, all of our patients declared that their self-injurious behaviours made them seek adequate medical treatment at least once before the moment of the study. We found that 91% of girls and 75% of boys declared that at least on one occasion it happened that they hurt themselves more severely than they expected. Table 9 illustrates the Practice patterns of the NSSI exhibited in the study group.

Table 10 shows the Perceived Life Interference caused by the NSSI.

The treatment experiences in relation to NSSI are shown in Table 11. In the group of teenagers that we studied, 85% of girls and 80% of boys declared going to a therapist at least once before our study. However, only 12.5% of the girls declared that they saw a therapist because of the self-harming behaviours; in the case of boys, half of them responded that they sought professional help for their self-injurious actions.

## 4. Discussion

In the final discussion of our research, we would like to focus on several aspects: the age of the studied group and their gender in relation to the self-harming behaviours, psychiatric comorbidities, and different characteristics of NSSI.

### 4.1. Age

In our study, we analysed NSSI in a sample of 100 Romanian adolescents hospitalized in the Child and Adolescent inpatient unit from January 2019 to November 2023. Regarding the nature of our study group, that consisted of hospitalized patients, we could hypothesize a particular representation of the self-harming phenomenon in a clinical sample. On the one hand, adolescents who engage in NSSI are unlikely to seek clinical help probably because of the stigma. On the other hand, teenagers might address mental health facilities looking for adequate support, while offering a positive significance to NSSI [1].

Anterior research indicates that approximately 23% of adolescents have reported intentionally harming themselves at least once in their lives, with nearly 19% doing so within the past year [18]. However, there is considerable variability in the prevalence of NSSI during adolescence [11]. Concerning the onset of this phenomenon, earlier studies suggested that self-harm typically begins between the ages of 11 and 15 [8]. However, a different study [9] found that NSSI peaks during adolescence, specifically between the ages of 15 and 17, and then declines or diminishes in late adolescence or early adulthood. While some research suggests that NSSI may commence before puberty [19], other studies suggest that it increases later in adolescence [20], with puberty being considered a critical period in the development of self-injury [18].

The data from our study is in concordance with data published so far, the mean age at NSSI onset, determined from NSSI-AT, in our group being 14.72 years (SD ± 1.87) for girls and, 15.2 years (SD ± 1.72) for boys.

We also considered it relevant to describe NSSI in this age category due to the fact that adolescents who engage in self-harm often come into contact with various healthcare providers, including paediatricians, school counsellors, psychologists, and psychiatrists. Research focused on self-harm in adolescents can provide valuable information to clinicians about assessment, diagnosis, and intervention strategies tailored to this population. Thus, selecting adolescents as the focus of a study on self-harm offers numerous benefits, including the high prevalence of self-harming behaviours during this developmental period, the developmental significance of adolescence, the identification of risk and protective factors, the long-term implications of self-harm, as well as the clinical relevance to healthcare providers.

### 4.2. Gender

According to anterior reports, the lifetime prevalence of self-harm is 4.3% for men and 13.5% for women [21], with some studies reporting a reversal in the ratio of NSSI prevalence between males and females, with increasing recognition of NSSI among males, particularly concerning the manner and frequency of behaviour activation, the severity and nature of associated psychopathology, and distinct cultural settings. Females may be more likely to engage in NSSI as a way to cope with emotional distress, regulate negative emotions, or seek interpersonal connection. In contrast, males may be more inclined to engage in NSSI as a means of demonstrating toughness or exerting control over their bodies. Gender differences may also exist in help-seeking behaviours among individuals who engage in NSSI. Research suggests that females are more likely to seek professional help or disclose their self-harming behaviours to others, while males may be more reluctant to seek help due to stigma or perceptions of weakness [22,23,24,25,26,27,28,29,30].

In our sample, there was a difference between the recruited number of female participants (*n* = 80) and the number of male participants (*n* = 20). There could be several reasons behind this imbalance. Firstly, the number of patients presenting in our clinic with NSSI during the studied period of time was 911—out of which 694 (76%) were female and 217 (24%)—male. Another reason comes from the fact that those who agreed to take part in this research were predominantly female. Thus, it could be argued that in our group there is a higher prevalence of NSSI in female patients than in male ones but these specifics should be kept in mind.

### 4.3. Psychiatric Comorbidities

When examining the relationship between psychiatric comorbidities and NSSI (Non-Suicidal Self-Injury), several important considerations emerge.

Research consistently shows that individuals who engage in NSSI often have high rates of psychiatric comorbidities. Common psychiatric disorders that frequently co-occur with NSSI include mood disorders (e.g., depression, bipolar disorder), anxiety disorders (e.g., generalized anxiety disorder, post-traumatic stress disorder), and personality disorders (e.g., borderline personality disorder) [29,30]. The relationship between psychiatric comorbidities and NSSI is often bidirectional, meaning that each can exacerbate the other. For example, individuals with pre-existing mood disorders may engage in NSSI as a maladaptive coping mechanism for managing intense negative emotions. Conversely, NSSI may also contribute to the development or worsening of psychiatric symptoms over time. Several underlying mechanisms may contribute to the association between psychiatric comorbidities and NSSI, such as deficits in emotion regulation, difficulties in interpersonal relationships, histories of trauma or adverse childhood experiences, and genetic predispositions to both NSSI and psychiatric disorders [30,31,32,33].

In our sample, NSSI was most common in patients with depressive disorders and anxiety-related pathologies, as well as disharmonious personality traits specific to personality disorders, with 54% of our patients having one or more comorbid psychopathologies.

We consider it of utmost importance to thoroughly evaluate the presence of psychiatric comorbidities in individuals who engage in NSSI, as it has important implications for treatment planning and intervention. Psychiatric comorbidities can also increase the risk of NSSI-related complications, including suicidal behaviour [34,35,36]. Integrated treatments that target both NSSI and co-occurring psychiatric disorders, such as dialectical behaviour therapy (DBT) or cognitive-behavioural therapy (CBT), may be particularly effective in reducing self-harm behaviours and improving overall mental health outcomes [37,38,39].

The relationship between psychiatric comorbidities and NSSI is complex and multifaceted, with implications for assessment, treatment, and risk management. Understanding and addressing psychiatric comorbidities is essential for providing comprehensive care to individuals who engage in NSSI and reducing the risk of adverse outcomes associated with self-harm behaviours.

### 4.4. NSSI Characteristics

In our study, we focused on the main emotional and behavioural components of NSSI and their relationship to different clinical NSSI phenotypes sorted by self-injury forms, self-injury functions, recency and frequency of NSSI, wound location, initial motivation, NSSI severity, practice patterns, perceived life interference, and NSSI treatment experiences.

Health professionals have long been concerned about NSSI, and clinical research is increasingly focusing on this topic [40,41,42]. Although one documented role of NSSI is protection from suicide, NSSI also serves as a risk factor for subsequent suicidal behaviour [13,40]. The Interpersonal Theory of Suicide can explain this relationship because, even in cases where NSSI serves as a temporary coping mechanism for suicidal thoughts, it may ultimately raise the risk of suicide through processes like increasing a person’s propensity to self-harm [41].

Numerous self-reported functions, such as emotional management, self-punishment, and distress communication, have been linked to NSSI [12,13]. Many individuals use NSSI as a way to self-regulate and cope with overwhelming or distressing emotions, such as sadness, anxiety, anger, or numbness. Self-harm may provide temporary relief from emotional pain or help individuals regulate intense emotions by providing a sense of control or distraction. Some individuals engage in NSSI as a form of self-punishment or self-blame, often in response to feelings of guilt, shame, or worthlessness. Self-harm may be used as a means of expressing self-directed anger or punishing oneself for perceived failures or shortcomings. For some individuals, NSSI serves as a way to communicate inner distress or communicate unmet needs to others. By engaging in self-harming behaviours, individuals may seek support from others, particularly when they feel unable to express their emotions verbally [37,38,39,40,41,42].

Individuals who endorse distinct underlying functions will require different kinds of assistance and interventions. Previous studies indicate that adolescents who self-harm exhibit a maladaptive coping loop later in life where emotions, thoughts, and self-harming behaviour reinforce one another [43]. Furthermore, Daukantaitė et al. showed that even in cases when teenagers used NSSI as a stress reliever on an irregular basis, they may still face adverse consequences in their early adult years [44].

In the last 5 years, in the studied group of 100 adolescent patients of an inpatient Romanian unit, the predominant form of self-harm observed was cutting, followed by hitting objects and burning. These behaviors primarily served the purpose of regulating emotions (“to feel something”, “to relieve stress or pressure”), or ”because they liked the way it looks” in the case of girls, while the main functions of self-harming for boys were represented by dealing with anger, managing discomforting emotions and converting emotional distress into physical sensations, thus focusing more on the emotional regulatory role of self-harm.

Regarding frequency, during their assessment, most male patients had engaged in NSSI between 11 and 50 times, while girls reported self-harming between 2 and 10 times. Concerning NSSI severity, all the subjects admitted to having harmed themselves more severely than anticipated on at least one occasion, necessitating specialized medical attention. Understanding NSSI frequency and severity is essential for comprehensive assessment, risk management, and treatment planning for individuals who engage in self-harming behaviours, as it provides valuable information about the patterns and trajectory of self-harm, guiding clinicians in providing appropriate evaluation, support, and care.

It is important to note that individuals may experience multiple functions of NSSI simultaneously or may engage in self-harm for different reasons at different times. Additionally, NSSI functions may evolve over time or in response to changes in individuals’ circumstances or psychological states. Understanding the functions of NSSI is crucial for developing effective interventions that address the underlying motivations driving self-harming behaviours and promoting healthier coping strategies.

In the studied group, the percentage of patients that saw a therapist before the moment of the admittance in our clinic, because of the self-harming behaviours was represented by 50% of the male participants and merely 12.5% of the female subjects. The treatment experience of every adolescent patient dealing with a mental health disorder is frequently complex and tumultuous, as each and every one of them needs an individual approach, catered to their particularities and specific needs. Seeking help and finding adequate therapeutic interventions often represents a major challenge for our patients, as a lot of them delay the initial moment of affirming their need for help.

## 5. Study Strengths and Limitations

We would argue that a descriptive study on self-harming behaviours such as the one we presented serves several important purposes and holds significant relevance in various contexts. Firstly, it provides valuable insights into the prevalence rates of self-harming behaviours within a specific population (psychiatric adolescent patients aged 13–17 years).

Secondly, it can help identify patterns related to the frequency, methods, onset and contexts of self-harm, which can inform prevention and intervention efforts. By examining demographic and environmental data of patients who practice self-harming behaviours, our study could contribute to the identification of risk factors that increase vulnerability to self-harm, and suggest future investigation of potential protective factors that mitigate risk. This knowledge could be used for targeted interventions and support strategies for at-risk individuals.

Moreover, considering the evaluation instrument and methods that were described, our study could contribute to the enhancement of other clinicians’ (from different medical specialties focusing on the health of children and adolescents), understanding of self-harming behaviours, facilitating more accurate assessment and timely diagnosis of such conditions. This could contribute to the development of tailored treatment plans and providing appropriate support and care for individuals experiencing self-harm, such as psychoeducation programs, skills training, cognitive-behavioural therapies, and access to mental health services, aimed at reducing self-harm and promoting healthier coping mechanisms.

While we consider that our study provides valuable insights on self-harming behaviours, it also has several limitations that we shall further point out.

The study has limited generalizability, as it focused on a specific population—psychiatric adolescent patients in a clinical setting. As a result, the findings may not be representative of broader populations or applicable to individuals outside the study sample, although we consider that focused research on this specific population can contribute to clinicians’ having a better insight on such behaviours in this category.

We should also consider that our data were gathered during a specific period of time, thus providing a snapshot of self-harming behaviours and associated factors. This cross-sectional design can limit the establishing of causal relationships or the examination of changes in self-harm over time, making it challenging to infer causality or predict long-term outcomes.

Another limitation resides in the confounding variables that could influence the relationship between variables of interest (for example—psychiatric comorbidities). Thus, in the absence of experimental control or statistical adjustment for confounders, alternative explanations for the observed behaviour characteristics cannot be ruled out.

Considering the characteristics of the NSSI-AT instrument, there is also a potential for recall bias. This measure relies on participants’ accurate recall and reporting of self-harming behaviours, which may be subject to recall bias. Individuals may underreport or overreport their self-harm experiences due to memory limitations, social desirability biases, or reluctance to disclose sensitive information, leading to inaccuracies in data.

In spite of providing information on the frequency, methods, initial motivation, functions and contexts of self-harming behaviours, this study does not capture detailed underlying psychological processes driving self-harm. Understanding the complex interplay of factors contributing to self-harming behaviours would require more in-depth qualitative research or mixed-method approaches.

## 6. Conclusions

This study investigated non-suicidal self-harm (NSSI) in a sample of 100 adolescents hospitalized for various psychiatric disorders, using the NSSI-AT instrument, in order to highlight differences in methods, NSSI functions and motivations between genders, as well as frequency and severity of these behaviours. From a clinical perspective, our findings shed a light on the various aspects of adolescent self-harm, providing information to be taken into account during specialised evaluation processes, as well as illustrating the need for targeted therapy interventions, tailored after these individual aspects.

Considering the potential public health implications, our research can contribute to the broader scientific understanding of self-harming behaviours in adolescents by documenting trends, exploring underlying functions and motivations for such behaviours, and generating hypotheses for further investigation. The results we presented could be used in order to raise awareness among different clinicians and specialists who interact with adolescents regarding the ever-increasing self-harming behaviours, so that adolescents who engage in such actions are directed to specialized evaluation. The need to raise awareness on this matter in our country rises from the fact that, although there are already numerous international studies focusing on non-suicidal self-harming behaviours, research on this matter in our country is limited. In Romania, there are still specialists (such as general practitioners, paediatricians, school doctors) who overlook the evaluation of self-harm in adolescents. A study on a Romanian group of adolescents could have a greater informational impact on a national level, and our results could be used to enrich the knowledge of these specialists.

In terms of future research directions, our study can provide a foundation for more complex research designs, such as longitudinal studies and experimental trials, for example those aimed at elucidating causal relationships and evaluating intervention efficacy, or exploring different NSSI aspects in more diverse populations.

## Figures and Tables

**Table 1:** Socio-demographic characteristics of the study group.

	Number	
Female patients with NSSI	80	
Male patients with NSSI	20	
Living area	Urban	Rural
	63 F	17 F
	12 M	8 M
Ethnicity	Caucasian—80 F; 20 M	
Age	Number	
13	27 (25 F, 2 M)	
14	18 (17 F, 1 M)	
15	15 (12 F, 3 M)	
16	18 (14 F, 4 M)	
17	22 (12 F, 10 M)	
Mean age	14.9 (SD ± 1.51)	
Age of onset (age at first NSSI incident) (years)		
	Female	Male
12	14	2
13	19	3
14	12	1
15	12	1
16	21	8
17	22	5
Mean age of onset	14.72 (SD ± 1.87)	15.2 (SD ±1.72)

**Table 2:** Clinical diagnoses of the study group.

Diagnosis	Number	
	Female	Male
Depressive disorders	100%	100%
Anxiety disorders (including obsessive-compulsive disorder)	59%	35%
Conduct disorders	16%	20%
Eating disorders	14%	0
Disharmonious personality traits	85%	80%

**Table 3:** NSSI Characteristics—Self-injury forms.

Self-Injury Forms	Number	
	Female	Male
Sustained significant scratching or pinching, potentially with fingernails or other objects, resulting in bleeding or lasting marks on the skin	72%	10%
Self-inflicted cuts on wrists, arms, legs, torso, or other body areas	100%	100%
Applied acid on skin	0	0
Incised words or other symbols onto the skin	16%	5%
Consumed corrosive substance(s) or sharp object(s)	0	0
Inflicted self-bites resulting in bleeding or skin markings	9%	5%
Attempted self-infliction of bone(s) fracture	1.25%	5%
Fractured one’s bone(s)	1.25%	5%
Torn or shredded skin	76%	45%
Sustained burns on wrists, hands, arms, legs, torso, or other regions of the body	40%	75%
Applied glass onto the skin or inserted sharp objects like needles, pins, and staples into or beneath the skin (excluding tattooing, body piercing, or medical needle use)	27%	50%
Struck or impacted objects until bruising or bleeding occurred	39%	80%
Struck or impacted oneself to the extent of bruising or bleeding	34%	40%
Deliberately hindered the healing process of wounds	64%	85%
Participated in physical fights or other aggressive actions with the aim of sustaining injury	2.5%	15%

**Table 4:** Self-injury functions.

Self-Injury Function	Number	
	Female	Male
Affective imbalance, low pressure		
To manage difficult emotions (e.g., depression, anxiety)	79%	85%
To transform emotional distress into a physical sensation	74%	80%
“To feel something”	89%	75%
To achieve a sense of agency or empowerment in their life	29%	45%
Social communication and expression		
In hopes that someone would notice that something is wrong or that so others will pay attention to them	19%	15%
To shock or hurt someone	21%	15%
Because their friends hurt themselves	14%	15%
Self-retribution and deterrence		
As a self-punishment or to atone for sins	12%	20%
Because of self-hatred	49%	35%
So that they do not hurt themselves other ways	26%	55%
To avoid committing suicide	23%	45%
Sensation seeking		
Because they felt the urge and could not stop it	70%	60%
Because it feels good	77%	80%
To get a rush or surge of energy	26%	10%
Because they liked the way it looks	83%	35%
Affective imbalance, high pressure		
To alleviate stress or pressure	86%	85%
To manage frustration	80%	80%
To address anger	66%	90%

**Table 5:** Recency and frequency of NSSI.

Recency	Number	
	Female	Male
Within the past month	89%	85%
Between 1–3 months ago	7%	15%
Between 3–6 months ago	4%	10%
Between 6 months to 1 year ago	0	0
Between 1 to 2 years ago	0	0
Over 2 years ago	0	0
Frequency		
Just one time	8%	10%
2–10 times	64%	40%
11–50 times	20%	45%
More than 50 times	7%	5%

**Table 6:** Wound locations.

Location	Number	
	Female	Male
Arms	91%	75%
Hands	14%	25%
Wrists	100%	100%
Thighs	54%	15%
Stomach or chest	19%	10%
Calves or ankles	59%	65%
Fingers	0	0
Head	0	0
Face	1%	10%

**Table 7:** Initial motivation.

Motivation	Number	
	Female	Male
A friend recommended that I give it a try	21%	10%
After reading about it online, I decided to experiment with it	28%	25%
I came across it in a movie, on television, or in a book, and decided to give it a shot	2.5%	0
It appeared to be effective for people I know	22%	15%
It appeared to be effective for celebrities I am familiar with	0	0
I stumbled upon it accidentally; I had no prior knowledge or exposure to it	27%	20%
It was part of a challenge	0	5%
I engaged in it because my friends were doing it, and I wanted to belong	19%	0
I desired to be part of a social circle	19%	0
I aimed to shock or cause harm to someone	28%	15%
I was distressed and opted to try it	89%	90%
I sought attention for myself and/or my injuries	25%	25%
It provided a sense of pleasure	84%	60%
I harbored resentment towards someone else	24%	35%
I was upset with myself	46%	55%
I was under the influence of alcohol or drugs	8%	30%
I have no recollection	11%	10%

**Table 8:** NSSI Severity.

Severity	Number	
	Female	Male
Have you ever inadvertently injured yourself more severely than anticipated?	91%	75%
Have you ever harmed yourself to such an extent that it warranted medical attention?	14%	25%
Have you ever sought medical assistance for any physical injuries resulting from non-suicidal self-injury (NSSI)?	100%	100%

**Table 9:** Practice patterns.

	Number	
	Female	Male
Social dimensions of NSSI practice		
I consistently inflict intentional harm upon myself in solitude	79%	80%
At times, I purposefully inflict harm upon myself while others are around	4%	10%
I have deliberately caused physical harm to another individual	2.5%	5%
Occasionally, I allow others to intentionally cause me physical harm	0	5%
Routines		
I experience cycles where I deliberately harm myself followed by periods where I refrain from doing so, and this cycle continues	84%	65%
I have a specific location or room where I prefer to be when I intentionally harm myself	80%	70%
I adhere to a consistent routine when I intentionally inflict harm upon myself	27%	10%

**Table 10:** Perceived Life Interference.

	Number	
	Female	Male
My deliberate self-harm poses a significant challenge in my life	91%	75%
My intentional self-harm disrupts:		
Relationships that hold significance to me	22.5%	55%
My capacity to fulfill academic or professional responsibilities	14%	35%
My ability to attend to self-care needs (such as nutrition, exercise, etc.)	14%	30%
My participation in hobbies or activities I enjoy	8%	25%
My sense of self-worth and self-esteem	39%	25%
My choice of clothing	76%	20%

**Table 11:** NSSI treatment experiences.

	Number	
	Female	Male
Ever gone to therapy		
Yes	85%	80%
No	15%	20%
Ever saught therapy for NSSI		
Yes	12.5%	50%
No	19%	30%
NSSI only part of the reason for therapy	55%	20%

## Data Availability

Restrictions apply to the availability of these data. Data was obtained from clinical interviews and from the database of “Prof. Dr. Al. Obregia” Clinical Psychiatry Hospital. Data from the hospital’s database were made available solely to the authors of the paper with the permission of the Hospital’s Ethics Committee.
